# Vomiting of Pregnancy

**Published:** 1891-02

**Authors:** 


### VOMITING OF PREGNANCY.

---

R Cerii oxalat., gr. j.
Ipecacuanhae, gr. j.
Creosoti, gtt. ij.

Sig. This is to be taken every hour until nausea is controlled.—*Prof. Goodell, Philadelphia.*—*Ex.*

